# Personality profiles in adults with attention deficit hyperactivity disorder (ADHD)

**DOI:** 10.1186/s12888-016-0906-6

**Published:** 2016-06-14

**Authors:** Nader Perroud, Roland Hasler, Nicolas Golay, Julien Zimmermann, Paco Prada, Rosetta Nicastro, Jean-Michel Aubry, Stefano Ardu, François R Herrmann, Panteleimon Giannakopoulos, Patrick Baud

**Affiliations:** Department of Mental Health and Psychiatry, University Hospitals of Geneva, Geneva, Switzerland; Department of Psychiatry, University of Geneva, Geneva, Switzerland; Section of Dental Medicine, Faculty of Medicine, University of Geneva, Geneva, Switzerland; Division of Geriatrics, Department of Internal Medicine, Rehabilitation and Geriatrics, Geneva University Hospitals and University of Geneva, Geneva, Switzerland

**Keywords:** ADHD, TCI, Attention, Hyperactivity, Impulsiveness, Personality

## Abstract

**Background:**

Previous studies suggested that the presence of ADHD in children and young adolescents may affect the development of personality. Whether or not the persistence of ADHD in adult life is associated with distinct personality patterns is still matter for debate. To address this issue, we compared the profiles of the Temperament and Character Inventory (TCI) that assesses personality dimensions in 119 adults ADHD and 403 controls.

**Methods:**

ANCOVA were used to examine group differences (controls vs. ADHD and ADHD inattentive type vs. ADHD combined + hyperactive/impulsive types) in Temperaments and Characters. Partial correlation coefficients were used to assess correlation between TCI and expression and severity of symptoms of ADHD.

**Results:**

High novelty seeking (NS), harm avoidance (HA) and self-transcendence (ST) scores as well as low self-directedness (SD) and cooperativeness (C) scores were associated with ADHD diagnosis. Low SD was the strongest personality trait associated with adult ADHD. Cases with the ADHD inattentive type showed higher HA and lower SD scores compared to the combined and hyperactive/impulsive types. High HA scores correlated with inattention symptoms whereas high NS and ST scores were related to hyperactive symptoms. Finally low SD and high NS were associated with increased ADHD severity.

**Conclusions:**

Distinct temperaments were associated with inattentive versus hyperactive/impulsive symptoms supporting the heterogeneous nature of the disorder.

## Background

Attention-deficit hyperactivity disorder (ADHD) is characterized by impaired attention control, impulsiveness and hyperactivity (motor restlessness). This disorder affects 3 to 7 % of children and persists in adulthood in roughly 30 to 60 % of subjects [1]. A deleterious familial environment in childhood as well as perinatal adverse events have been frequently reported in adult ADHD cases and are thought to have a negative impact on personality features [2]. Earlier studies in this field revealed that adults with ADHD tend to be more pessimistic, introverted, rebellious, and aggressive [3], show high levels of psychological distress, dissatisfaction, low capacity to be organized and self-disciplined, and are less sociable, altruistic and sympathetic to others [2, 4].

Among the tools used to assess personality, the Temperament and Character Inventory (TCI), a self-report questionnaire assessing four dimensions of temperament (novelty seeking (NS), harm avoidance (HA), reward dependence (RD) and persistence (P)) and three dimensions of character (self-directedness (SD), cooperativeness (C) and self-transcendence (ST)), based on the Cloninger’s biopsychosocial theory of personality, has aroused growing interest in ADHD subjects [5]. Its development was based on the assumption that personality construct can be described as a combination of a small number of temperament and character dimensions. Reflecting basic emotional responses to external and/or internal stimuli, temperaments are moderately heritable, and fairly stable throughout life [6, 7]. They have been hypothesized to play a role in the development of psychopathology in several psychiatric disorders including ADHD [8, 9].

Characters are believed to represent more complex cognitive processes and refer to individual differences in goals, self-concepts and values. Being tightly linked to environmental influences, character dimensions may vary as a function of developmental stages during lifespan.

Previous studies on temperaments in adults and children suffering from ADHD led to highly comparable data showing high scores on NS, conceived as the tendency to be impulsive and irritable versus rigid and stoical, high scores on HA, defined as the tendency to be pessimistic and anxious versus optimistic and risk-taking, low scores on P, assessing the tendency to be persevering and ambitious versus easily discouraged and indolent, and low scores on RD defined as the tendency to be sociable and warm versus aloof and cold [4, 10–21].

In terms of character patterns, earlier data reported that ADHD subjects differ from controls by low SD, referring to the tendency to be blaming and inept, low C associated with hostility and opportunism, and high ST describing people who are intuitive and insightful [10–12, 15–18, 21, 22]. Most of these temperament and character changes in ADHD subjects support the idea of low psychological functioning [16].

However, ADHD is not a clinically homogeneous condition. In recent years, three main subtypes (according to DSM-V) have been recognized, the predominantly inattentive presentation if the subject mainly has symptoms of inattention, the predominantly hyperactive-impulsive presentation if mainly symptoms of hyperactivity-impulsivity are present, and the combined presentation where both criteria of inattention and hyperactivity-impulsivity are present. To date, several studies have investigated which personality dimension is associated with specific subtypes of the disorder however leading to controversial data [10, 13, 15, 17, 20, 23–26]. Overall these studies linked deviation in NS to hyperactive/impulsive symptoms and deviation in HA to inattentive symptoms although deviation in almost all of the temperaments and characters have been reported.

Some studies using the Five Factor Model (FFM) of Personality also investigated the link with the severity of ADHD, but this has been poorly explored using the TCI leading to conflicting data [27]. For instance, Salgado et al. [13] found that the combined subtype of ADHD was associated with higher scores in NS and lower scores in C and HA than the predominantly inattentive subtype. They also found an inverse relationship between the number of inattentive symptoms and SD scores and a positive correlation with HA scores, whereas the number of hyperactive/impulsive symptoms were positively correlated with NS and P scores. Lynn et al. [15] found that NS and the character dimension C accounted for the majority of variability in symptoms of hyperactivity-impulsivity while inattention was mainly predicted by NS. Faraone et al. [17] found that hyperactive/impulsive and inattention symptoms positively correlated with NS and HA scores and negatively with RD, P, SD and C scores. Gomez et al. [23], in a population based study of ADHD, found association between inattention and HA and between hyperactive/impulsive symptoms and P. Wood et al. [24] observed in children a genetic correlation between NS and hyperactive/impulsive symptoms. In adolescents, Young et al. [25] showed that attention and hyperactive/impulsive symptoms were mainly explained by NS. Van Dijk et al. [20] in a study comparing subjects suffering from borderline personality disorder to ADHD subjects, found that high NS was associated with the inattention symptoms of ADHD. In a twin study, Merwood et al. [26] found that NS was genetically associated with both symptoms of inattention and hyperactivity/impulsivity and that HA was genetically associated with inattention only. They also found some evidence of genetic association between P and the two ADHD domains (inattention and hyperactivity/impulsivity) suggesting that different profiles of temperament are genetically linked to ADHD dimensions in adults. Purper-Ouakil et al. [10] also found evidence showing that temperaments and characters were related to specific symptoms of ADHD. In their non-clinical sample of children, attention problems were negatively correlated with SD whereas NS was related to externalized symptoms and less to attentional problems. C was also inversely correlated with externalized symptoms. In the clinical sample (ADHD subjects) NS was linked to impulsivity/hyperactivity whereas SD (low) was associated with inattention.

In conclusion, although concordant results emerged when comparing controls to ADHD subjects, this is not the case when looking at the different subtypes and severity of ADHD. In this perspective, we explored the association between ADHD subtypes and temperament and characters in adult life as well as that between these personality patterns and severity of the disease in a large community-based sample of adult ADHD cases compared to healthy controls. Based on the above-mentioned literature, we were expecting to find high NS, HA and ST scores as well as low SD and C in subjects suffering from ADHD. We also hypothesized that deviation in NS and in HA would be related to hyperactive/impulsive and inattentive symptoms respectively. Finally, we were expecting to find an association between low SD and C scores with severity of ADHD.

## Methods

### Participants

One hundred and nineteen outpatients with adult ADHD were recruited in a specialized center for the diagnosis and care of adult ADHD patients at the University Hospitals of Geneva. ADHD diagnosis was made according to the DSM-IV criteria based on a clinical interview with trained psychiatrists (NP, PP, JZ and PB). Six or more symptoms of inattentive and/or hyperactive-impulsive symptoms were required and should be present before 7 years of age. The number of symptoms was used to determine the ADHD subtype as described by Prada et al. [28]. Briefly, a subject with 6 or more inattentive symptoms but less than 6 hyperactive-impulsive symptoms was classified as predominantly inattentive; a subject with 6 or more hyperactive-impulsive symptoms but less than 6 inattentive symptoms was classified as predominantly hyperactive-impulsive; and a subject with 6 or more hyperactive-impulsive and inattentive symptoms was classified as combined type.

Most of the adult ADHD patients (N = 89) were addressed by either general practitioners or psychiatrists for an initial assessment and for a suspicion of ADHD and were thus free of any psychopharmacological treatment at the time of their recruitment; although some of them previously received antidepressants for previous major depressive disorder. The remaining 30 subjects were already diagnosed with ADHD and were, for most of them, taking psychostimulants when reaching our tertiary center.

The comparison group included 403 subjects recruited from the general population in the Blood Donor Center of the University Hospitals of Geneva and School of Dentistry at the University of Geneva.

Controls and ADHD patients with neurological conditions such as epilepsia were excluded from the study.

The study was approved by the ethics committee of Geneva University Hospitals. Informed written consent was obtained from all participants.

### Instruments

A semi-structured interview assessing childhood and adulthood ADHD based on DSM-IV criteria: “Entretien diagnostique pour le TDAH chez l’adulte” (DIVA) [29] as well as the French version of the Diagnostic Interview for Genetic Studies (DIGS) [30] were administered to ADHD patients. The DIGS not only assesses Axis I comorbidities but also ADHD symptoms in childhood and their persistence into adulthood. In addition, all of the ADHD subjects completed the Wender Utah Rating Scale (WURS) [31], a self-report questionnaire assessing the severity of childhood ADHD, and the Adult ADHD Self-Report Scale (ASRS v1.1) [32], which assesses the severity of adult ADHD. They also fulfilled the Childhood Trauma Questionnaire (CTQ),which examines five types of trauma (sexual abuse, physical abuse, physical neglect, emotional abuse and emotional neglect) to assess childhood traumatic experience [33].

Finally, ADHD subjects completed the TCI. The TCI is a 240-items (235 research items and 5 validation items for the assessment of response accuracy and carelessness) self-report questionnaire evaluating temperament and character dimensions. Each question should be answered by yes or no, assessing the four dimensions of temperament (novelty seeking, harm avoidance, reward dependence and persistence) and the three dimensions of character (self-directedness, cooperativeness and self-transcendence). Besides the 7 main scales, the TCI also provides a number of subscales (3 to 5) for each of the temperaments and characters (which will not be taken into account in the current paper). The Cronbach’s alpha was good for each of the TCI subscales with values comprised between 0.89 and 0.93.

All of the controls completed the TCI. Among them, 191 subjects were also screened for Axis I disorders for main diagnosis and comorbidities (142 negative), using the Mini International Neuropsychiatric Interview [34].

### Statistics

One-way analysis of covariance (ANCOVA) with adjustment for age, included as a continuous was used to compare the Temperaments’ and Characters’ means between ADHD and controls.

Given the high frequency of psychiatric comorbidities in adults suffering from ADHD, and in order to test a "real life" hypothesis, two analyses were performed. The first concerned the whole sample: ADHD subjects without the exclusion of psychiatric comorbidities and all controls. In this analysis, controls corresponded to the notion of non-ADHD. The second analysis was made in ADHD subjects without psychiatric comorbidities and controls after exclusion of psychiatric disorders.

ANCOVA was also used to compare the Temperaments’ and Characters’ means between ADHD with the predominantly inattentive type and ADHD with the two other types (hyperactive/impulsive + combined). Given the small number of subjects suffering from the predominantly hyperactive/impulsive ADHD subtype (n = 8), these subjects were pooled with subjects suffering from the combined subtype (N = 65) for statistical purposes.

Finally, within ADHD subjects, partial correlation coefficients were computed between TCI temperaments and characters and ADHD symptoms, and severity with adjustment on age and gender. Partial correlation coefficients is calculated between one given variable (ex: NS) and a variable after removing the effects of all other variables entered into the model.

As characters are believed to be highly influenced by environmental factors, and thus vary during different stages of lifespan, we wondered whether childhood maltreatment would be associated with characters. Partial correlation coefficients were thus used to assess strength of correlation between different types of childhood maltreatments and TCI temperaments and characters within ADHD subjects.

When data was not normally distributed (namely HA, SD, ST, and P) Box-Cox transformation was used to take the appropriate remedial actions. This procedure helps to identify an appropriate exponent (Lambda = l) to use to transform data into a “normal shape.” The power to which all data should be raised is determined by the Lambda value. To do so, the Box-Cox transformation seeks from Lambda = −5 to Lamba = +5 until the best value is found. Then the appropriate action to transform the data is taken. STATA SE v.12.0 was used for the analyses and p value of ≤0.05 was considered as significant.

## Results

Table 1 shows the TCI characters and temperaments of ADHD and control subjects. Compared to controls, ADHD diagnosis was associated with higher NS scores (F_(1/521)_ = 28.3; p < 0.0001), higher HA scores (F_(1/521)_ = 54.2; p < 0.0001), higher ST scores (F_(1/521)_ = 9.2; p = 0.003), lower SD scores (F_(1/521)_ = 269.7; p < 0.0001) and lower C scores (F_(1/521)_ = 26.4; p < 0.0001). Similar results were obtained (same magnitude of effect size) after exclusion of psychiatric comorbidities in ADHD subjects and exclusion of psychiatric disorders in controls (Table 1). Moreover, adding marital status as an environmental factors that may influence characters into the models did not change the results.

**Table 1:** Panel A: Comparison of demographic and clinical characteristics of ADHD and control subjects for the whole sample; Panel B: Comparison of demographic and clinical characteristics of ADHD without Axis I comorbidities and control subjects without psychiatric disorders. For dimensional variables, ANCOVA were done with adjustment on age. TCI-R (Temperament and Character Inventory)

		A. Whole Sample						B. Subjects without Axis I comorbidity(ADHD)/disorder(Controls)					
		ADHD (*n* = 119)		Controls (*n* = 403)		ADHD vs. Controls		ADHD (*n* = 49)		Controls (*n* = 142)		ADHD vs. Controls	
		*N*	%	*N*	%	OR	*p*	*N*	%	*N*	%	OR	*p*
Female		37	31.1	148	36.7	1.29	0.26	15	30.6	38	26.8	0.83	0.6
		Mean	SD	Mean	SD	F(df)	*p*	Mean	SD	Mean	SD	F(df)	*p*
Age		37.3	11	45.5	12.7	40.6(1/521)	<0.0001	35.6	12.8	45.5	13.2	28.8(1/260)	<0.0001
TCI-R	NS	23	5.9	18.8	5.6	28.3(1/521)	<0.0001	23.1	5.4	18.9	5.1	19.4(1/260)	<0.0001
	HA	18.3	8.1	12.7	6.2	54.2(1/521)	<0.0001	16.5	8.5	11.1	5.5	50.4(1/260)	<0.0001
	RD	14.2	4	14.6	3.8	2.8(1/521)	0.096	13.7	3.5	14.3	4	0.6(1/260)	0.458
	P	4.8	2	4.8	2	0.03(1/521)	0.863	4.8	2.2	4.7	2	0.02(1/260)	0.892
	ST	15.3	6.8	13.5	6.3	9.2(1/521)	0.003	15.1	7.2	13	6.3	8.4(1/260)	0.004
	SD	21.9	8.5	34.5	5.8	269.7(1/521)	<0.0001	23.4	8.7	35.3	5.4	161.4(1/260)	<0.0001
	C	30.5	7.4	33.5	5	26.4(1/521)	<0.0001	30.1	6.1	33.7	4.5	14(1/260)	0.0002

*Abbreviations*: *NS* Novelty Seeking, *HA* Harm Avoidance, *RD* Reward Dependence, *P* Persistence, *ST* Self-Transcendence, *SD* Self-Directedness, *C* Cooperativeness

### Comparison of personality patterns in ADHD subtypes

The ADHD predominantly inattentive type was related to lower ASRS v1.1 and WURS scores (Table 2). ADHD of the predominantly inattentive type was associated with lower NS scores and higher HA scores (F_(1/118)_ = 9.3; p = 0.003 and F_(1/118)_ = 5.2; p = 0.024) (Table 2). Adding Axis 1 comorbidity (yes or no) as covariate led to similar results (data not shown).

**Table 2:** Clinical and demographic characteristics of ADHD subtypes

		ADHD predomin. inattentive (*N* = 46)		ADHD comb. and h/i types (*N* = 73)		Comparisons	
		*N*	%	*N*	%	OR	*p*
Gender	Female	14	30.4	23	31.5	1.05	0.902
Main Axis I comorbidity	Schizoaff. Dis.	1	2.2	1	1.4	0.96	0.741
	Subst. Use dis.	0	0	1	1.4		
	Bip. Dis.	0	0	9	12.3		
	Maj. Dep. Dis./Dyst.	24	52.2	34	46.6		
	No	21	45.7	28	38.4		
		Mean	SD	Mean	SD	F(df)	*p*
Age		39.3	12.2	36	10.1	2.6(1/118)	0.112
WURS		46.4	14.4	56.8	16.9	11.9(1/118)	0.0007
ASRS_v1.1		44.5	9.9	50.6	10	10.3(1/118)	0.002
Age at onset of ADHD		6	2.2	5.4	1.6	3.2(1/118)	0.077
TCI	NS	21	6	24.3	5.5	9.3(1/118)	0.003
	HA	20.3	7.2	16.9	8.4	5.2(1/118)	0.024
	RD	14.6	4	14	4	0.8(1/118)	0.365
	P	4.7	2.2	4.8	1.9	0.2(1/118)	0.655
	ST	14.3	6.8	15.9	6.8	1.6(1/118)	0.209
	SD	21.4	7.7	22.3	9	0.3(1/118)	0.592
	C	32.3	6	29.3	8	4.7(1/118)	0.033

*Abbreviations*: *NS* Novelty Seeking, *HA* Harm Avoidance, *RD* Reward Dependence, *P* Persistence, *ST* Self-Transcendence, *SD* Self-Directedness, *C* Cooperativeness, *TCI* Temperament and Character Inventory, *WURS* Wender Utah Rating Scale, *ASRS v1.1* Adult ADHD Self-Report Scale

### TCI correlation with ADHD symptoms and severity

Table 3 shows the results of the partial correlation coefficients considering TCI temperaments and characters and ADHD symptoms and severity. Inattention symptoms positively correlated with HA and negatively with SD whereas hyperactive symptoms positively correlated with NS and ST. SD and C negatively correlated with the severity of ADHD in childhood. The severity of current ADHD was positively related to NS, RD, and ST and negatively correlated with SD.

**Table 3:** A Partial correlation coefficients* between TCI temperaments and characters and number of ADHD symptoms. B. Partial correlation coefficients* between TCI temperaments and characters and continuous scales measuring severity of ADHD. Results with p < =0.05 are bolded and italicized

	NS	HA	RD	P	ST	SD	C
	Coeff; *p*	Coeff; *p*	Coeff; *p*	Coeff; *p*	Coeff; *p*	Coeff; *p*	Coeff; *p*
A.							
Inattention symptoms	0.05; 0.55	***0.27; 0.003***	0.14; 0.12	−0.14; 0.13	0.10; 0.28	***−0.25; 0.007***	−0.13; 0.15
Hyperactive symptoms	***0.21; 0.02***	−0.17; 0.06	−0.01; 0.88	−0.08; 0.39	***0.18; 0.05***	0.06; 0.48	−0.08; 0.41
Impulsive symptoms	−0.07; 0.44	−0.04; 0.65	−0.04; 0.67	0.16; 0.06	−012; 0.20	0.11; 0.24	0.01; 0.97
B.							
WURS score	−0.02; 0.86	0.09; 0.35	−0.11; 0.25	−0.04; 0.63	0.07; 0.45	***−0.31; 0.0008***	***−0.27; 0.003***
ASRS-v1.1 score	***0.23; 0.01***	0.11; 0.25	***0.21; 0.02***	−0.02; 0.82	***0.19; 0.03***	***−0.20; 0.03***	0.001; 0.90

*Abbreviations*: *NS* Novelty Seeking, *HA* Harm Avoidance, *RD* Reward Dependence, *P* Persistence, *ST* Self-Transcendence, *SD* Self-Directedness, *C* Cooperativeness

*Partial correlation coefficients is calculated between one given variable (ex: NS) and a variable after removing the effects of all other variables entered into the model

### TCI correlation with childhood maltreatment

None of the different types of childhood maltreatment did significantly correlate with TCI temperaments and characters (Table 4).

**Table 4:** Partial correlation coefficients* between TCI temperaments and characters and different types of childhood maltreatments after adjustment on age and gender

	NS	HA	RD	P	ST	SD	C
	Coeff; *p*	Coeff; *p*	Coeff; *p*	Coeff; *p*	Coeff; *p*	Coeff; *p*	Coeff; *p*
Emotional neglect	−0.15;0.12	0.14;0.12	−0.13;0.16	0.11;0.22	−0.07;0.41	−0.04;0.60	−0.10;0.26
Emotional abuse	−0.01;0.97	−0.07;0.41	−0.01;0.87	−0.04;0.67	−0.02;0.83	−0.05;0.59	−0.05;0.56
Physical neglect	0.10;0.29	0.06;0.51	0.01;0.96	−0.06;0.46	0.13;0.15	−0.04;0.61	0.01;0.98
Physical abuse	−0.03;0.72	0.01;0.95	−0.16;0.09	0.12;0.19	0.11;0.23	−0.01;0.91	−0.02;0.79
Sexual abuse	0.02;0.81	0.02;0.48	0.15;0.11	−0.15;0.11	0.13;0.14	0.08;0.63	0.12;0.18

*Abbreviations*: *NS* Novelty Seeking, *HA* Harm Avoidance, *RD* Reward Dependence, *P* Persistence, *ST* Self-Transcendence, *SD* Self-Directedness, *C* Cooperativeness

## Discussion

In agreement with our hypotheses, our data revealed striking differences in both temperament and character dimensions between adult ADHD and healthy controls. We indeed found that high NS, HA and ST scores as well as low SD and C scores were associated with ADHD diagnosis. We also found that high HA scores helped to distinguish the predominantly inattentive type from the pooled combined-hyperactive/impulsive subjects and that NS positively correlated with hyperactive symptoms. Finally, as expected, low SD scores were associated with increased ADHD severity.

Confirming previous investigations in this field [10, 11, 35–37], low SD and C scores were associated with ADHD diagnosis in the present series. SD reflects the individual’s ability for autonomy, his/her reliability and maturity, how responsible, purposeful and resourceful he/she is and thus how this individual is able to achieve his/her own goal and to be autonomous [38]. Low scores on SD have been shown to predict personality disorders mainly from the cluster B and in particular borderline personality disorder [16, 20]. In line with this idea, some authors have suggested that ADHD may represent a core vulnerability factor for borderline personality disorder in adolescence [39–42]. C indicates how well an individual is able to get along with others and closely reflects interpersonal skills such as empathy, compassions or the ability to be helpful. In addition to the lower SD, low C may thus relate to a deficit in maturity of personality and social skills and thus low psychological functioning and poor mental health outcomes [10, 18, 37, 43–45]. Interestingly, SD negatively correlated with the severity of ADHD in adulthood and childhood and, concordant with previous findings, a negative correlation between C and severity of ADHD in childhood was also found [11, 15, 35]. In this perspective, Martel et al. found that, in children, low C partly mediated the effect of genetic factors on ADHD symptoms with a main impact on the inattentive subtype [45]. Taken together, these results indicate that character immaturity is associated with ADHD severity possibly leading to the paucity of interpersonal relationships observed in severe forms of this disorder [11, 13, 37]. Whether this association reflects early neurodevelopmental deficits is still unclear in the absence of longitudinal data.

Not surprisingly, high NS scores were also found in ADHD subjects compared to controls [12, 14–16, 18, 37, 46]. However, and in contrast to previous studies, this association was modest in our cases [14, 15]. NS mainly reflects individual variability in initiating a behavior in response to stimuli and comprises impulsiveness, the search for novel and unknown experiences, but also spontaneity and extraversion in social situations. According to Cloninger [47], people with high NS are impulsive, curious, disordered, extravagant, easily bored and hot-headed, which is, by definition, a trait expected to be higher in ADHD subjects than in controls. As previously reported, NS positively correlated with ADHD severity and hyperactive symptoms [11, 13, 16, 19, 23, 26]. High NS scores are indeed rather related to impulsivity/hyperactivity rather than inattentive problems [19, 26]. It is thus not surprising to find that, in our study, NS enabled to distinguish the predominantly inattentive type from the pooled combined-hyperactive/impulsive subjects with the latter displaying the highest scores.

Several previous reports found higher HA in ADHD compared to controls [4, 14–17, 35, 37]. Our results were consistent with these findings showing that higher HA was associated with ADHD. This association persist after controlling for psychiatric comorbidities such as anxiety, depression and substance use disorders [12, 18]. Our findings suggest that ADHD subjects are, independently of other psychiatric comorbidities, easily worried, fearful, shy, socially reserved, and easily tired [1, 48]. In addition, higher HA scores enabled to distinguish the predominantly inattentive type from the pooled combined-hyperactive/impulsive subjects [13, 23]. In the same line, recent research showed a robust association between HA and inattentive symptoms, primarily due to overlapping genetic influences [26]. As suggested by Merwood et al., this association may be suggestive of an increased risk for internalizing symptoms such as anxiety disorders in ADHD subjects with higher number of inattentive symptoms [26, 49].

Last but not least, higher ST scores were found in ADHD subjects compared to controls. Moreover, ST positively correlated with ADHD severity in adulthood. In agreement with previous reports, these results support the idea that ADHD subjects tend to be spiritual, satisfied, modest and self-forgetful [17, 18]. Intriguingly, high ST scores have been found in several psychiatric entities suggesting that ST may be a vulnerability trait for neuropsychiatric disorders. In the Cloninger’s theory, high ST combined with low scores on the two other characters is associated with schizotypal and paranoid symptoms and may provide a good proxy for the existence of a personality disorder [50]. It has to be kept in mind that spirituality and religion may help patients to cope with their illness. If this has been more deeply investigated for disorders such as schizophrenia, this has not been investigated in relation to ADHD and further researches are needed in this field [51].

Although previous studies found low RD in ADHD subjects [17], suggesting that they are socially insensible and indifferent, our results did not corroborate this viewpoint. ADHD subjects scored roughly the same as controls on RD indicating that they did not differ from the general population in terms of attachment and dependence.

This study has several limitations, starting with the relative small sample size of ADHD subtypes. Secondarily, this study relies on self-report questionnaires assessing personality traits that are hypothesized to reflect the individual’s characters and temperaments throughout the course of his/her life. This hampers the possibility of determining the temporal relationship between personality changes and ADHD occurrence. Thirdly, only a fraction of our controls were assessed for psychiatric disorders and comorbidities, and thus some of them may still suffer from ADHD or other psychiatric disorders that have not been identified. Our separate analysis showed that the exclusion of psychiatric comorbidities in both groups did not alter the observed associations. Moreover, more that 70 % of the screened controls did not have any psychiatric disorder further decreasing the probability of erroneous assumptions due to comorbidities. In addition, we did not assess several other clinical and demographic variables potentially affecting TCI dimensions and more specifically characters that are believed to be highly associated with environmental influences. We were thus not able to adjust for such variables and this caveat should be kept in mind when interpreting our findings. Finally, we did not control for the fact that some ADHD patients took psychostimulants in childhood and that this may have affected the development of their personality.

## Conclusions

The present observations suggest that ADHD is associated with specific personality traits which may reflect poor mental health outcomes. Our results are concordant with previous contributions using the Five Factor Model (FFM) of personality showing that ADHD subjects whatever is their age display deviation in several personality dimensions [27]. In a recent and elegant meta-analytic review, Gomez and Corr have shown, using the integrated FFM proposed by Markon et al. [52] which includes the personality dimensions of several questionnaires including the FFM and the TCI, that ADHD subjects compared to controls have low levels of conscientious inhibition which may be interpreted as high level of NS, low levels of agreeable inhibition (roughly corresponding to C), and high levels of negative emotionality which includes the TCI temperament HA and is inversely correlated to SD. As mentioned above, determining whether characters and temperaments are risk factors for the development of ADHD or the opposite deserves further investigation. However temperaments have been constructed as biological dimensions emerging early in life and thus possibly before the onset of ADHD. This may not be the case for characters that often reflect the influence of ADHD on personality development. Therefore, assuming that temperaments precede the onset of ADHD, targeting NS in childhood may provide an interesting predictor for identifying at risk ADHD subjects.

Our results also reveal that HA and NS scores may differentiate inattentive from hyperactive/impulsive ADHD subtypes [44, 53, 54]. Following Merwood et al., we may propose that the individual’s profile of temperaments may help identify ADHD subtypes possibly better than relying only on a clinical evaluation [26].

In conclusion, our study offers a compelling picture of personality deviations in ADHD and how they relate to symptom dimensions. Longitudinal studies are clearly needed in this field in order to better understand the complex interplay between personality and ADHD symptoms.

## Abbreviations

ADHD, Attention-deficit hyperactivity disorder; ANCOVA, One-way analysis of covariance; ASRS, Adult ADHD Self-Report Scale; C, cooperativeness; CTQ, Childhood Trauma Questionnaire; DIGS, Diagnostic Interview for Genetic Studies; DIVA, Entretien diagnostique pour le TDAH chez l’adulte; FFM, Five Factor Model; HA, harm avoidance; NS, High novelty seeking; P, persistence; RD, reward dependence; SD, self-directedness; ST, self-transcendence; TCI, Temperament and Character Inventory; WURS, Wender Utah Rating Scale.
